# A systemic macrophage response is required to contain a peripheral poxvirus infection

**DOI:** 10.1371/journal.ppat.1006435

**Published:** 2017-06-14

**Authors:** Michael L. Davies, Nikhil J. Parekh, Lauren W. Kaminsky, Chetna Soni, Irene E. Reider, Tracy E. Krouse, Matthew A. Fischer, Nico van Rooijen, Ziaur S. M. Rahman, Christopher C. Norbury

**Affiliations:** 1 Department of Microbiology and Immunology, College of Medicine, Pennsylvania State University, Hershey, PA, United States of America; 2 Department of Molecular Cell Biology, Faculty of Medicine, Vrije Universiteit, BT Amsterdam, The Netherlands; University of Wisconsin-Madison, UNITED STATES

## Abstract

The goal of the innate immune system is to reduce pathogen spread prior to the initiation of an effective adaptive immune response. Following an infection at a peripheral site, virus typically drains through the lymph to the lymph node prior to entering the blood stream and being systemically disseminated. Therefore, there are three distinct spatial checkpoints at which intervention to prevent systemic spread of virus can occur, namely: 1) the site of infection, 2) the draining lymph node via filtration of lymph or 3) the systemic level via organs that filter the blood. We have previously shown that systemic depletion of phagocytic cells allows viral spread after dermal infection with Vaccinia virus (VACV), which infects naturally through the skin. Here we use multiple depletion methodologies to define both the spatial checkpoint and the identity of the cells that prevent systemic spread of VACV. Subcapsular sinus macrophages of the draining lymph node have been implicated as critical effectors in clearance of lymph borne viruses following peripheral infection. We find that monocyte populations recruited to the site of VACV infection play a critical role in control of local pathogenesis and tissue damage, but do not prevent dissemination of virus. Following infection with virulent VACV, the subcapsular sinus macrophages within the draining lymph node become infected, but are not exclusively required to prevent systemic spread. Rather, small doses of VACV enter the bloodstream and the function of systemic macrophages, but not dendritic cells, is required to prevent further spread. The results illustrate that a systemic innate response to a peripheral virus infection may be required to prevent widespread infection and pathology following infection with virulent viruses, such as poxviruses.

## Author summary

Prior to the eradication of variola virus, the orthopoxvirus that causes smallpox, one-third of infected people succumbed to the disease. Despite many complications, smallpox vaccination using vaccinia virus enabled a successful eradication of the disease. Following smallpox eradication, vaccinia (the smallpox vaccine) remains a widely used vaccine vector, so any information about the immune response to the vector can help engineer safer vaccines, or treatment, following complications of immunization. During natural infection, orthopoxviruses spread from a peripheral site of infection to become systemic. This study elucidates the early requirement of innate immune cells to control spread of the smallpox vaccine vector after a peripheral infection. We report that systemic populations of cells, rather than those recruited to the site of infection, are responsible for preventing virus dissemination. The viral control mediated by these cell subsets presents a potential target for therapies and rational vaccine design.

## Introduction

A large number of viruses infect the host at the periphery and spread systemically through the lymphatic system to cause disease. This is the same mechanism by which many viruses of concern to human and animal health such as orthopoxviruses (variola virus, monkeypox virus), enteroviruses (polio, coxsackie), Aphthovirus (foot-and-mouth disease), Rubivirus (rubella), Flavivirus (Yellow Fever, Dengue, West Nile), Rubulavirus (mumps), Morbillivirus (measles), Varicelovirus (chickenpox), and others, spread and cause disease [1, 2]. When a pathogen breaches the epidermis, an ideal innate immune response attacks the infectious agent and keeps the infection localized to the initial site of inoculation, so the host does not risk a fulminant, disseminated infection. Here, we investigate the cellular mechanisms responsible for preventing widespread dissemination following dermal virus infection.

A number of potential checkpoints exist to stop or blunt the spread of virus following peripheral infection. Recruitment of innate immune cells, such as neutrophils or monocytes/macrophages, to the site of infection (in this case, the skin) could restrict or slow the spread of virus. However, cellular recruitment can take hours to days so a rapidly replicating virus could spread prior to migration of innate immune cells to the site of infection. After inoculation, infectious virus quickly enters the lymphatic system and empties into the draining lymph nodes (D-LN). Particles carried by lymph first enter the subcapsular sinus (SCS) of a D-LN where they are taken up by CD169^+^ SCS macrophages, [3]. Infection of SCS macrophages may be vital to prevent the spread of virus and is important for efficient activation of the immune system. SCS macrophages are optimized for virus uptake and antigen presentation to B cells, fulfilling a function during peripheral viral infection that is analogous to the role of metallophilic marginal zone (MZ) macrophages in the spleen during viremia [4]. CD169^+^ macrophages in LN and spleen may even support limited replication of some viruses, which can be important for providing sufficient viral antigen to quickly activate antiviral immunity [4–7]. If not internalized by SCS macrophages, virus may be internalized by or infect less specialized macrophages in the medullary sinuses [8] (akin to the splenic MZ macrophages that border the red pulp). If both of these populations of macrophages are absent, inactive, or overwhelmed, the assumption is that virus may enter the bloodstream, allowing a systemic infection [9, 10].

Systemic macrophage populations that are in close contact with the bloodstream, particularly those in the MZ of the spleen, but also in the liver or kidney, are targets of many bloodborne viruses [11–18]. Infection of MZ macrophages is thought to be important for production of Type-I interferon (IFN) [18], IL-1 [14], induction of T cells [16], or antibody [5] responses. The MZ macrophages also they function to “soak up” bloodborne virus to prevent additional spread [15]. In addition, myeloid cell populations in other organs, such as the liver, may also “soak up” bloodborne virus [12, 19–24]. However, none of the published data have utilized a manner of targeting macrophages within a specific organ without bystander effects upon other organs. Therefore, macrophages at the site of infection, in the D-LN, and in the splenic MZ or other blood rich organs may all play a role in controlling the systemic spread of virus, but the relative contributions of macrophage populations at each checkpoint following peripheral virus infection have not been assessed. Here, we will dissect the role of macrophages at different spatial checkpoints following dermal infection with the orthopoxvirus Vaccinia virus (VACV).

Poxviruses are a group of viruses that infect primarily through the skin (e.g. cowpoxvirus, monkeypoxvirus, and ectromelia virus (ECTV), which causes mousepox). A poxvirus that is currently endemic in the human population is molluscum contagiosum virus (MCV), the third most prevalent viral skin infection worldwide [25]. VACV is a poxvirus that can establish localized skin infection in a wide range of mammals and is widely used as a backbone for viral vaccine vectors. VACV is most effective as a vaccine when delivered via damage to the epidermis [26], and as a pathogen is adapted to the skin, as revealed by a number of viral immunomodulatory molecules that facilitate virus replication only during skin infection [27]. VACV remains localized to the skin in mice with intact immune systems [28]. However, if VACV does reach internal organs it replicates profusely, particularly in the ovaries of female mice [29], a pattern of tropism for epithelial and steroidogenic cells that resembles more virulent poxviruses [30]. This makes VACV an ideal, relevant model to study the factors that determine whether a peripheral infection will remain peripheral or become disseminated, as individual components of the immune system can be ablated and the spread of VACV measured. Monocytes migrate to the site of VACV infection in the skin [31, 32] and become infected with VACV at this site [33]. In addition, we [34], and others [33] have shown that VACV draining from the skin preferentially infect SCS macrophages in the D-LN. However, replicating VACV primarily remains restricted to the skin after dermal infection, displaying a relatively mild, localized pathogenesis [28] that may be related to the fact that VACV replicates poorly, if at all, in macrophages or DC, either murine or human [35–37]. In fact, both VACV and MCV infections are notable for the large numbers of skin-resident DC that leave the skin and migrate to the D-LN, but this migration of infected DC does not help the virus disseminate [38–40]. Other studies have suggested that infected monocytes [41] or neutrophils [42] may fulfill a “Trojan horse” role during dermal VACV infection. However, we previously established that mice depleted of phagocytes allow VACV dissemination from skin to the internal organs, spread that would not be possible if phagocytes were the primary carriers of the virus [32].

In our previous work we used multiple depletion methods to examine the role of myeloid cell populations in spread of VACV following dermal infection. Although we found that each manipulation of myeloid cell populations had profound effects upon local pathology in the skin following dermal VACV infection, only clodronate-loaded liposomes (CLL) administration allowed the spread of large quantities of VACV from the ear to the ovaries, the primary site of VACV replication [32]. Therefore, CLL uniquely depleted a population, or a combination of populations, of cells that are required to prevent systemic VACV spread. CLL relies upon the phagocytic property of a cell to internalize toxic liposomes, leading to cell death. In contrast, other mechanisms of myeloid cell depletion target either cells in which specific promoters are active, or cells with certain proteins displayed upon the cell surface. Therefore, the mechanism of targeting by CLL is unique, and CLL may deplete populations that are not depleted by other cell-depletion methodologies. Here, we expand upon our previous work by using additional methodologies to deplete myeloid cell populations, none of which replicate the ability of CLL to allow systemic spread of VACV, to identify the spatial checkpoint(s) at which VACV spread is contained.

In this study we use two complementary genetic mouse models to study the transient depletion of myeloid cells, primarily macrophages and neutrophils, during VACV infection. Both models utilize a system in which expression of a suicide receptor is driven by a “lineage-specific” promoter, and ligation of that receptor can allow ablation of the population expressing the receptor. First, we use MaFIA (Macrophage Fas-Induced Apoptosis) mice, which express a suicide receptor driven by the CD115 promoter [43]. Injection of the drug AP20187 causes dimerization of the suicide receptor and induction of apoptosis in cells in which the CD115 promoter, which drives expression of the M-CSF receptor, is or has been active. As a second approach, we use LysMcre:iDTR mice, which express a high-affinity diphtheria toxin (DT) receptor in cells that express *Lyz2* (the gene for lysozyme 2) [44–46]. Administration of DT depletes cells in which the Lyz2 promoter is or has been active, primarily granulocytes, but also monocytes, macrophages, and alveolar type II cells. Each of these systems allows effective and transient depletion of the target populations. Each of these promoters was chosen due to activity in particular myeloid cell populations, but use of a single promoter to target cell populations often means that other “off-target” populations may be depleted. In particular, both the Lyz2 and CD115 promoters have been described to be active in subpopulations of DC [47–49].

In order to control for the effects of the MaFIA and LysMcre:iDTR mouse models upon DC populations we used two additional models. In CD11ccre:iDTR mice, the expression of the DT receptor is driven by the CD11c promoter, that drives expression of an integrin subunit that is primarily active within DC. Thus, DT administration to CD11ccre:iDTR mice allows transient deletion of DC, as well as other cells that express CD11c (**Table 1**). In addition, we used Batf3^-/-^ mice, which have a germline deletion for the Batf3 transcription factor. Batf3^-/-^ mice lack the CD8^+^ subset of DC, which we have previously shown to be important in the innate immune response to ectromelia virus (ECTV), a virus related to VACV that is the causative agent of mousepox [50].

**Table 1:** Depletion methodologies during VACV infection.

Method	1^O^ target at site of infection	1^O^ target in D-LN	1^O^ target in systemic organs	Off target effects
**Systemic CLL**	Partial depletion of monocytes, macrophages	Some medullary macrophages and monocytes	MZ and other macrophages, monocytes	DC, especially CD8+ DC
**Local CLL**	Monocytes, macrophages	SCS and medullary macrophages	None	DC, especially CD8+ DC
**MaFIA**	Monocytes, macrophages	ND	ND	DC, Tissue Protective Ly6G+ cells
**LysMcre:iDTR**	Monocytes, macrophages and any other CD11b+ myeloid cells	Monocytes, SCS macrophages, neutrophils, CD8+ DC	Poor depletion of Monocytes, macrophages, neutrophils, CD8+ DC	DC, neutrophils, Tissue Protective Ly6G+ cells
**CD11ccre:iDTR**	Langerhans cells, monocyte-derived DC	CD11b+ and CD8+ DC, monocyte-derived DC	CD11b+ and CD8+ DC, monocyte-derived DC	Activated CD8+ T cells, some NK cells
**Batf3-/-**	None	CD8+ DC	CD8+ DC	ND
**Anti-Ly6G Ab**	Tissue Protective Ly6G+ cells	Neutrophils	Neutrophils	Activated CD4+ and CD8+ T cells
**CCR2-/-**	Monocytes	Monocytes	Monocytes	NK cells and some T cells
**CX3CR1-/-**	Poor depletion of monocytes	Poor depletion of monocytes	Poor depletion of monocytes	Some T cells

ND = Not Done

In order to target cell populations with certain proteins displayed on the cell surface we also used antibody-mediated depletion to remove populations of neutrophils (anti-Ly6G) and T cells (anti-Thy1 (CD90)). In each case we titrated doses of antibody carefully to ensure that depletion was >90% effective at all sites examined (site of infection, D-LN and spleen). Antibody remains in the injected mouse for a number of days, allowing continual depletion. However, again, the use of a single antigenic target allows depletion of unintended cell populations that may express that target. In this case, some T cell subsets express Ly6G and some keratinocytes and epithelial cells express Thy1. Therefore, care must be taken when interpreting results obtained following antibody depletion.

A further mechanism used to investigate the role of myeloid cells is genetic deletion of chemokine receptors required for a particular cell population to reach its intended site of action. An example used in this study is mice lacking the chemokine receptor CCR2, which is required for monocyte precursors to leave the bone marrow and enter the circulation, as well as for recruitment of inflammatory monocytes to some sites of inflammation [51]. Another example is mice lacking CX3CR1, a chemokine receptor expressed by resting monocytes (and many other cells, including T lymphocytes), which binds a ligand released from sites of inflammation. In CX3CR1^-/-^ mice the “circulating” or “patrolling” monocytes are unable to reach sites of tissue damage where they may be needed to replenish tissue-resident macrophages [52]. The disadvantage of using such chemokine receptor knockouts is that they have global effects, and rarely target individual populations of cells, potentially giving a global developmental defect that must be considered when interpreting experimental results.

Each of these mechanisms of examining the role of individual myeloid cell populations has advantages and disadvantages, but it is clear that a global perspective must be taken when interpreting results, rather than reliance upon a single depletion methodology (**Table 1**). Here we use multiple methodologies to gain a comprehensive picture of the role of myeloid cell populations in preventing the systemic spread of VACV following a peripheral infection. In using multiple methodologies we can eliminate the off target effects of any single approach. This is particularly important because the efficiency and specificity of these cell depletion methodologies have been established in the steady, or non-infectious disease, state. VACV infection induces a significant c-kit-dependent alteration in the hematopoietic stem cell compartment of the bone marrow, as myeloid cell precursors are released into the circulation [53] where they can produce atypical myeloid cells at the site of infection [32]. Therefore, an in-depth study of the effects of myeloid cell depletions or deficiency in the context of a VACV infection is warranted. Here, we utilize these depletion or deficiency models compared to CLL treatment to examine which cell populations at which anatomical location are required to prevent VACV spread following dermal infection. We find, surprisingly, the local macrophage populations do not play an exclusive role in preventing VACV dissemination. Rather, systemic macrophages are infected with bloodborne VACV and are depleted uniquely by CLL, indicating that these cells play a role in preventing systemic spread of VACV following dermal infection.

## Results

### Clodronate-loaded liposome (CLL) depletion allows VACV spread only early after dermal infection

**Fig 1:**
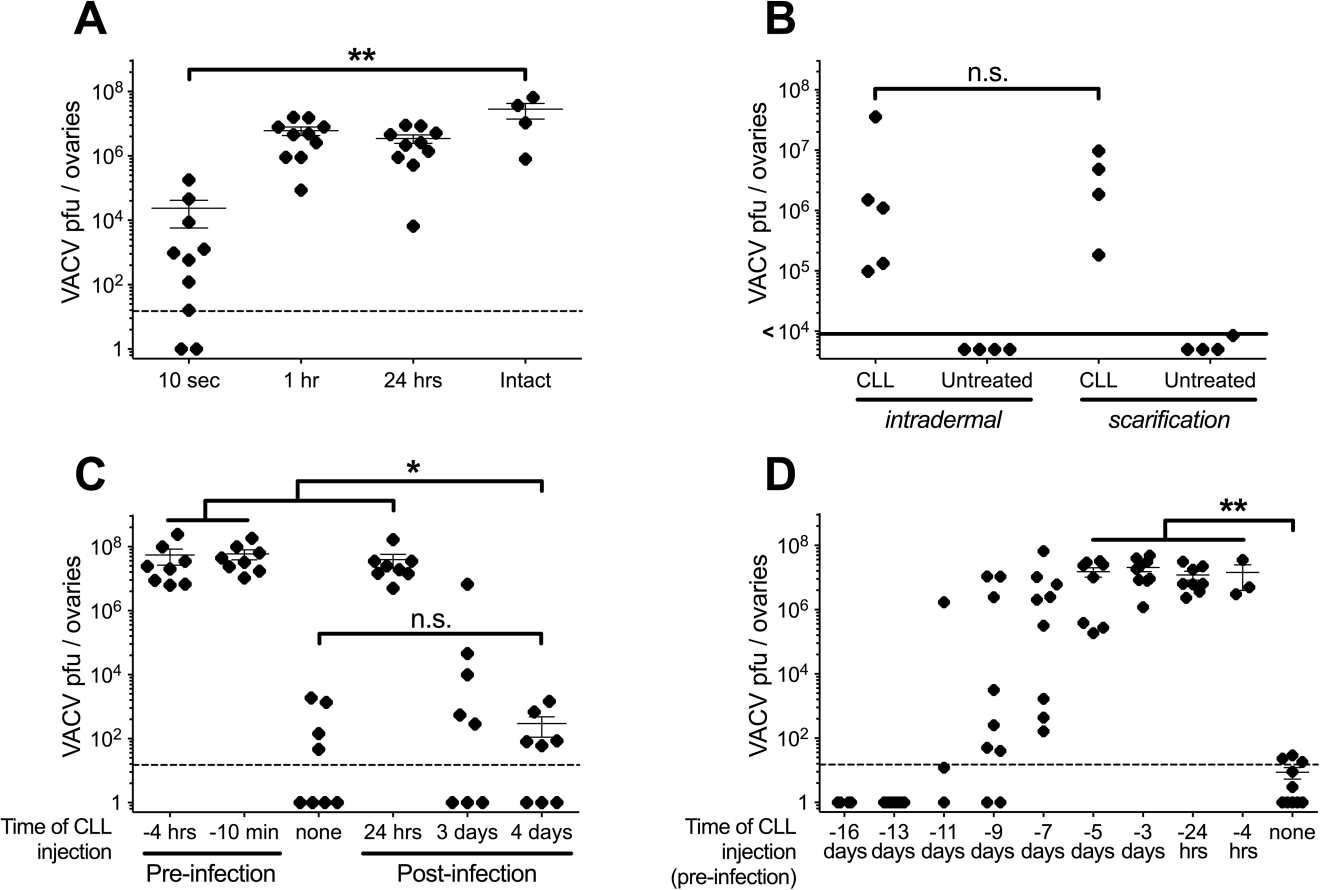
Kinetics of VACV spread and cell populations following intradermal VACV infection. C57BL/6 mice were infected intradermally with 10,000 pfu VACV in each ear pinna. **(A)** Mice were given a single injection of CLL *i*.*v*. 4 hours pre-infection, and their ear pinnae was surgically removed at the times shown post-infection. 5 days post-infection, ovaries were harvested and used in a plaque assay for VACV titer. **(B)** Mice were treated with a single injection of CLL *i*.*v*. 24 hours pre-infection via intradermal infection or scarification of the ear in the presence of 10^6^ pfu VACV. **(C)** Mice were given a single injection of CLL *i*.*v*. at the time shown pre- or post-infection. 5 days post-infection, ovaries were harvested and used in a plaque assay for VACV titer. **(D)** Mice were given a single injection of CLL *i*.*v*. at the time shown pre-infection. 5 days after infection, ovaries were harvested and used in a plaque assay for VACV titer. Results include all data from a minimum of 3 independent experiments (n = 8–14).

In our previous studies on mice examining intradermal VACV infection, we observed that systemic depletion of phagocytes with clodronate-loaded liposomes (CLL) allowed the virus to spread from the ears, becoming a disseminated infection detectable at high levels in internal organs [32]. To gain an insight into the mechanism by which phagocytes prevented VACV spread, we initially investigated at which point VACV could establish a systemic infection. To do this, we utilized the peripheral nature of our model to remove the initial site of infection at various times after inoculation. Mice were given a dose of CLL *i*.*v*. and then infected with 10^4^ pfu of VACV in the center of each ear pinna. The site of infection was removed at different times post-infection. Five days post-infection, the level of virus replication in the ovaries was similar whether the ear pinnae had been left intact for 1 hour, 24 hours, or the entire 5 days post-infection (**Fig 1A**). Even when ear pinnae were removed 10 seconds post-infection, some virus drained from the skin and later reached the ovaries, although not as much as if the pinnae were left intact for an hour or more (**Fig 1A**). This shows that a small intradermal inoculum of VACV is sufficient to establish disseminated infection within an hour. As CLL-depleted mice reliably showed titers in the ovaries on the level of 10^8^ VACV pfu/mouse, in subsequent figures we chose to display extremely low values as “< 10^4^ pfu”, the amount of the inoculum. We were concerned that the injection of a bolus of fluid intradermally may allow VACV to bypass natural checkpoints involved in preventing spread. Therefore, we compared the ability of CLL treatment to allow the systemic spread of VACV following intradermal infection to VACV administered to skin damaged by scarification. Systemic CLL treatment allowed spread of VACV, irrespective of whether the virus was infected *i*.*d*. or via scarification (**Fig 1B**). Therefore, the ability of CLL treatment to allow systemic spread of VACV does not depend upon injection of fluid to bypass immune checkpoints.

To examine when CLL-depleted cells are required to prevent disseminated infection, we infected mice intradermally and gave them a single dose of CLL *i*.*v*. at different times pre- or post-infection. We then determined the level of VACV replication in the ovaries 5 days post-infection. CLL given on the day of infection, or the next day, allowed VACV to spread to the ovaries (**Fig 1C**). Although virus was detectable in the ovaries of some untreated mice, the levels of VACV were ~4 orders of magnitude lower than those seen with CLL depletion (**Fig 1C**). However, by 4 days post-infection VACV spread following CLL administration was reduced over 10,000 fold compared to mice treated with CLL just prior to infection (**Fig 1C**). Therefore, despite the intensive virus replication occurring in the skin 4 days after infection, with VACV titers peaking at day 5 [32, 41], depletion of phagocytes only allowed virus to drain from the ear and establish systemic infection for 3 days post-infection. This finding indicates that the initial VACV inoculum, and replicating VACV at early time points following infection, can disseminate throughout the body in the absence of phagocytes depleted by systemic CLL treatment.

After establishing that the day of primary infection is the crucial moment for viral dissemination, we investigated how long phagocytes responsible for blocking VACV spread are absent after CLL depletion. We gave mice a single dose of CLL *i*.*v*., then infected with VACV at times ranging from 4 hours to 16 days post-depletion and assayed for the presence of VACV in the ovaries 5 days post-infection. Mice infected 5 days post-depletion still had uncontrolled dissemination of VACV to ovaries, and even at 7–11 days post-depletion not all mice were able to block virus dissemination (**Fig 1D**). Since VACV is still able to disseminate 24 hours post-infection, this indicates that it takes an absolute minimum of 6 days to reconstitute the crucial phagocyte population that prevents virus spread.

### Local depletion of myeloid cell populations increases local tissue damage but not systemic virus dissemination

**Fig 2:**
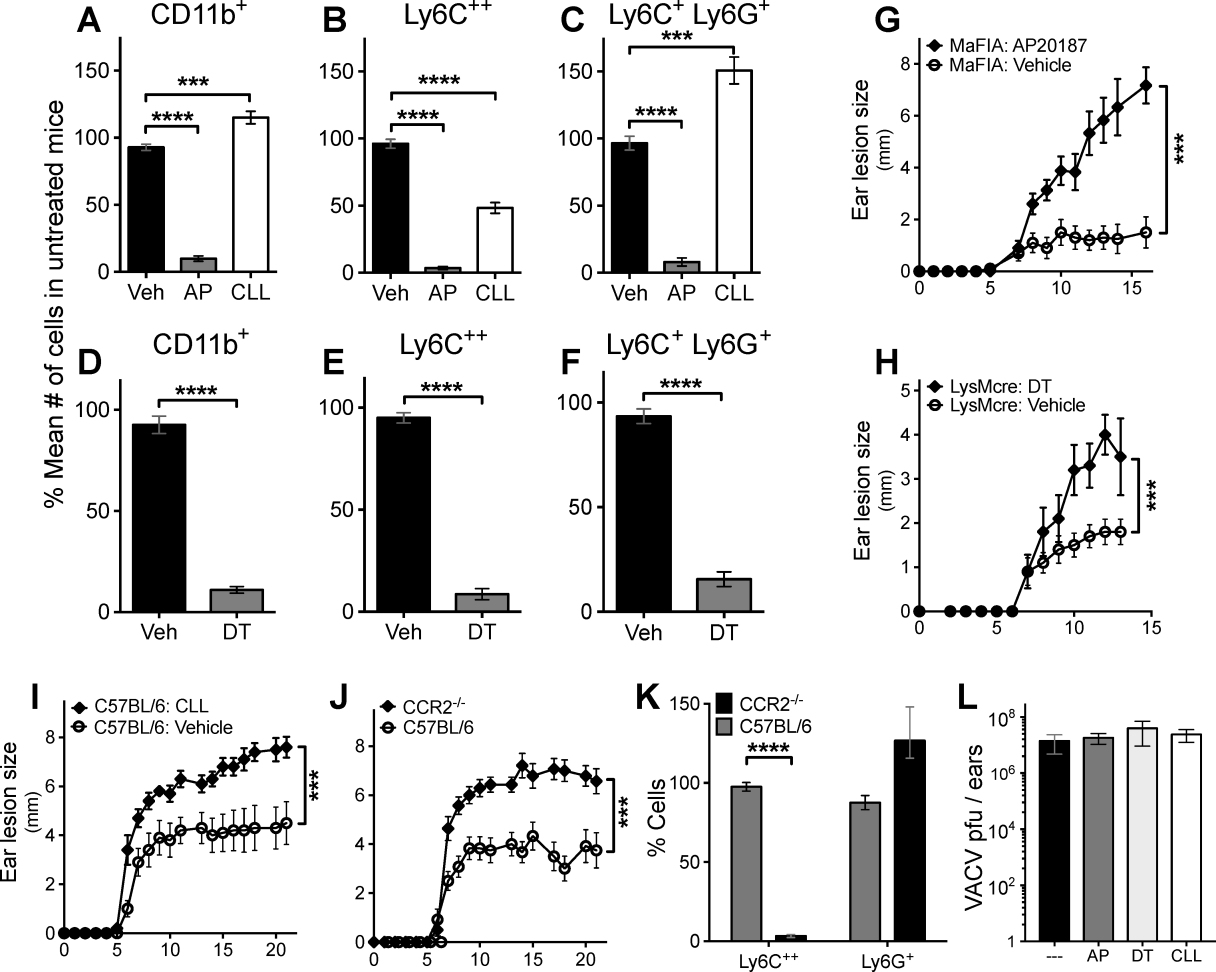
Depletion of local myeloid cell populations increases local pathogenesis and tissue damage. Mice on the C57BL/6 background were infected intradermally with 10,000 pfu VACV in each ear pinna. **(A-C)** MaFIA mice (n > 8) were injected with CLL i.v. as described in Fig 1A or AP20187 i.p. on Day 0 pre-infection and Days 1, 3 and 4 post-infection. At 5 dpi, ear pinnae were isolated and cells extracted. Flow cytometry was used to count the number of CD11b^+^ Ly6C^++^ Ly6G^-^ inflammatory macrophages, CD11b^+^ Ly6C^+^ Ly6G^+^ tissue-protective myeloid cells, and total CD11b^+^ myeloid cells. (**D-F)** LysMcre:iDTR mice (n = 4) were injected i.p. with DT (40 ng/g) or vehicle on the day of infection. At 5 dpi, ear pinnae were isolated and cells extracted. Flow cytometry was used to count the number of CD11b^+^ Ly6C^++^ Ly6G^-^ inflammatory macrophages, CD11b^+^ Ly6C^+^ Ly6G^+^ tissue-protective myeloid cells, and total CD11b^+^ myeloid cells. (**A**-**F**) In order to compile data across many experiments, data are expressed as % of the mean number of cells in untreated mice. Results include all data from a minimum of 3 independent experiments (n = 8–14). **(G-J)** Mice were treated as follows and VACV lesion size measured daily. **(G)** MaFIA mice (n = 5) were injected with AP20187 or vehicle as described in Materials and Methods. **(H)** LysMcre:iDTR mice (n = 5) were injected with DT (40 ng/g) i.p. or vehicle on Day 0 pre-infection and Days 2, 5, 8, 11 and 14 post-infection. **(I)** C57BL/6 mice (n = 5) were injected with CLL or vehicle i.v. on Day 0 pre-infection and Days 1, 3, 4, 11 and 18 post-infection. **(J)** CCR2^-/-^ or C57BL/6 mice were infected with VACV as above. All VACV pathogenesis data is representative of 3 independent experiments. **(K)** At 5 dpi, ear pinnae were isolated and cells extracted from C57BL/6 or CCR2-/- mice. Flow cytometry was used to count the number of CD11b^+^ Ly6C^++^ Ly6G^-^ inflammatory macrophages and CD11b^+^ Ly6C^+^ Ly6G^+^ tissue-protective myeloid cells. (**L**) C57BL/6 (treated or untreated with CLL), MaFIA or LysMcre:iDTR mice were infected as above. Ears were harvested 5 days post-infection and virus was quantified using a standard plaque assay.

To examine the role of phagocytic cells at the site of infection, we sought to establish conditions that deplete myeloid cell populations in the ear following dermal VACV infection. We first examined the efficacy of two depletion methodologies at removing CD11b^+^ phagocytes at the site of infection. First, we administered AP20187 to MaFIA mice to target cells that express, or have expressed, CD115 [43]. Cells were extracted from VACV-infected ear pinna at a time when inflammation and viral replication are still increasing (day 5 of infection), and analyzed by flow cytometry. When total myeloid cells were counted (FSC^Hi^ CD11b^+^ CD90.2^-^ CD19^-^ NK1.1^-^), AP20187 treatment eliminated the vast majority of myeloid cells (**Fig 2A**). We have previously characterized the infiltration of classical inflammatory monocytes (CD11b^+^Ly6C^++^ Ly6G^-^) and a distinct population of atypical CD11b^+^Ly6C^+^ Ly6G^+^ “tissue-protective” monocytes to the site of VACV infection [32]. MaFIA mice treated with AP20187 before and during infection saw a 95% decrease in recruited macrophages or inflammatory monocytes (**Fig 2B**) and a 90% decrease in Ly6G^+^ cells (**Fig 2C**). In contrast, CLL treatment was much less effective at reducing local macrophage numbers (**Fig 2B**) and increased numbers of both total CD11b^+^ cells and tissue-protective Ly6G^+^ cells (**Fig 2A and 2C**). As a second approach we administered diphtheria toxin (DT) to LysMcre:iDTR mice. We found that DT administration in LysMcre:iDTR mice reduced the number of CD11b^+^ phagocytes to a similar degree as AP20187 treatment in MaFIA mice (**Fig 2D**). Additionally, inflammatory macrophage (**Fig 2E**) and Ly6G^+^ cell (**Fig 2F**) populations were reduced >80% in the VACV-infected ear. Therefore, both the MaFIA and LysMcre:iDTR mouse models provide mechanisms to examine the role of locally recruited phagocytes relative to CLL treatment.

As the MaFIA and LysMcre:iDTR mouse models were substantially more efficacious at depleting local myeloid cell populations in the infected ear, we first examined the local pathogenesis caused by VACV infection. After intradermal infection with VACV and sustained cell depletion, the MaFIA and LysMcre:iDTR mouse models had larger skin lesions than mock-depleted littermates (**Fig 2G and 2H**). We have previously demonstrated that depletion of Ly6G^+^ cells causes a similar increase in lesion size in VACV-infected mice [32]. Therefore, we initially attributed the enhanced tissue damage to depletion of these tissue-protective cells in MaFIA and LysMcre:iDTR mice. However, we also observed markedly enhanced lesions in VACV-infected mice treated with CLL (**Fig 2I**), indicating that the Ly6C^++^ inflammatory macrophages at the site of infection can also modulate the pathogenesis following VACV infection. To specifically ablate the role of Ly6C^++^ inflammatory macrophages at the site of infection, we used CCR2^-/-^ mice in which inflammatory monocytes are completely ablated from the site of VACV infection (**Fig 2K**). In CCR2^-/-^ mice as well, the lesion size was considerably enhanced compared to wild-type mice (**Fig 2J**). However, the recruitment of Ly6G^+^ cells In CCR2^-/-^ mice was unaffected (**Fig 2K**), indicating that classical inflammatory macrophages as well as Ly6G^+^ myeloid cells [32] are needed to limit local tissue damage following VACV infection. Damage at the site of infection, and subsequent spread beyond the ear, could be affected by alterations in local VACV replication. We have previously shown that neither CLL nor AP20187 treatment have a large impact upon local VACV replication [32]. Here we expand that observation to show that DT treatment of LysMcre:iDTR also did not dramatically increase or decrease VACV replication locally (**Fig 2L**), also indicating that changes in local tissue pathogenesis may be due to changes in the immune response, rather than VACV replication.

**Fig 3:**
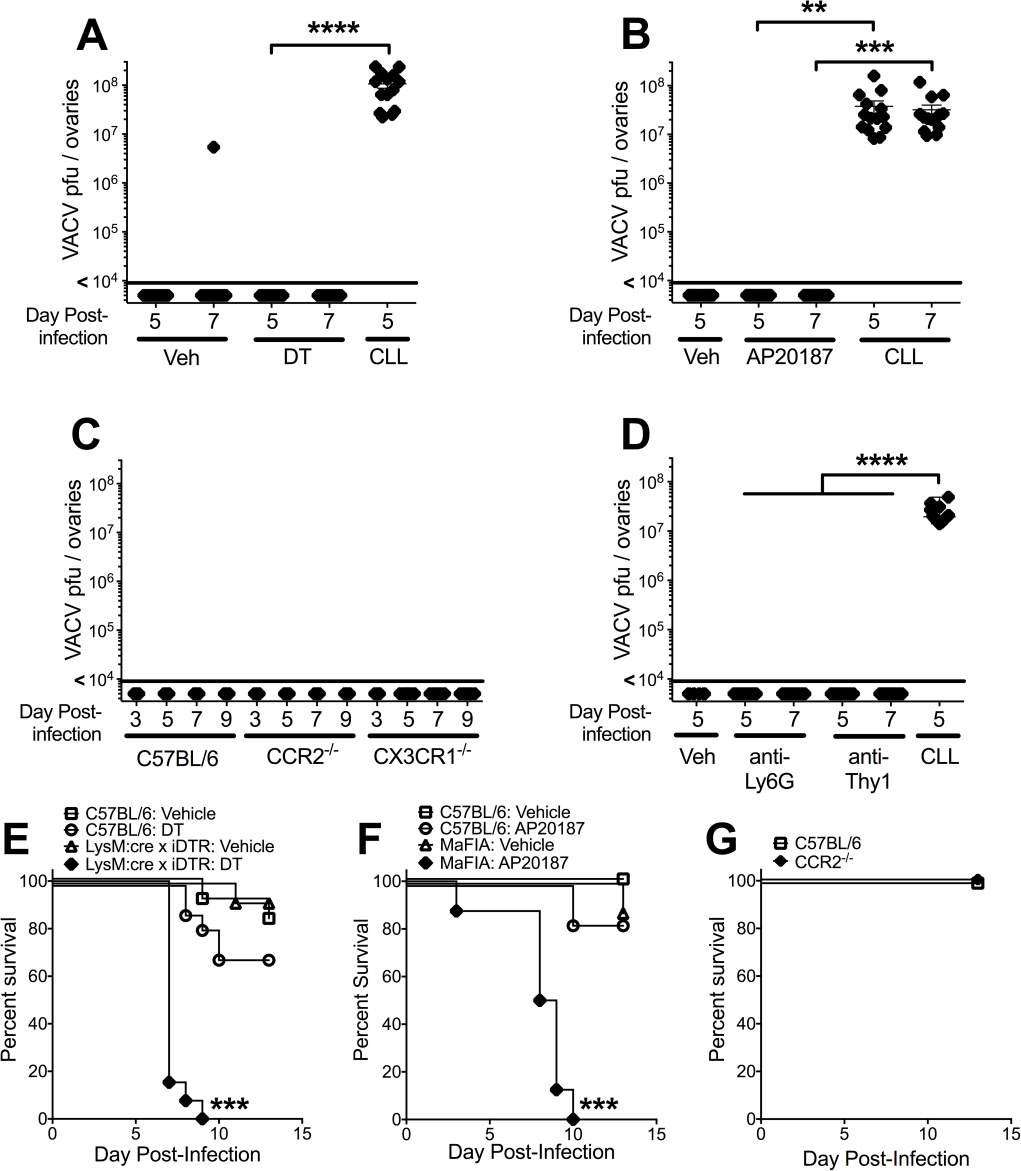
Depletion of local myeloid cell populations does not allow systemic spread of VACV, but does affect survival following ECTV infection. Mice (n ≥ 5) on the C57BL/6 background were infected intradermally with 10,000 pfu VACV in each ear pinna. (A) LysMcre:iDTR mice were injected with CLL i.v., DT (40 ng/g) i.p., or vehicle i.p., on day 0 pre-infection and days 1, 3 and 4 post-infection. At 5 or 7 dpi, ovaries were harvested and used in a plaque assay for VACV titer. Results include all data from a minimum of 3 independent experiments (n = 8–14). (B) MaFIA mice were injected with CLL i.v. on Day 0 pre-infection and Days 1, 3 and 4 post-infection, or AP20187 as described in Materials and Methods. At 5 or 7 dpi, ovaries were harvested and used in a plaque assay for VACV titer. Results include all data from a minimum of 3 independent experiments (n = 8–14). (C) Ovaries were harvested from wild-type, CCR2^-/-^, or CX3CR1^-/-^ mice 3, 5, 7 or 9 dpi and used in a plaque assay for VACV titer. Results include all data from a minimum of 3 independent experiments (n = 8–12). (D) Wild-type C57BL/6 mice were injected with CLL i.v. on Day 0 pre-infection, or the anti-Ly6G antibody 1A8 (50 μg/g) on Days -4, -2 and 0 pre-infection, or the anti-Thy1 antibody T24 (30μg/g) on day -1 pre-infection and 3 dpi. At 5 dpi, ovaries were harvested and used in a plaque assay for VACV titer. Results include data from 3 independent experiments (n = 9). (E-G) Mice were infected in the footpad with 3,000 pfu ECTV, and survival was monitored for 2 weeks post-infection. (E) LysMcre:iDTR or wild-type mice were injected i.p. with 40 ng/g DT or vehicle on days -3, -2, and -1 pre-infection and days 2, 5, 8 and 11 post-infection. (F) MaFIA mice or wild-type mice were injected i.p. with 10 μg/g AP20187 or vehicle as described in Materials and Methods. (G) C57BL/6 or CCR2^-/-^ mice were left undepleted and survival was monitored. All ECTV graphs represent pooled data from 2 to 4 experiments (n = 10–20).

Inflammatory macrophages recruited to the site of VACV infection clearly affect local pathogenesis, so we next sought to examine the ability of these cells to block the systemic dissemination of VACV following dermal infection. As before, we found that targeting phagocytes with CLL allowed virus dissemination from the site of infection and massive replication (10^7^–10^8^ pfu/mouse) in the ovaries (**Fig 3A**). However, targeting myeloid cells for depletion in LysMcre:iDTR mice treated with DT did not allow systemic spread of VACV, as replicating virus was not detected in the ovaries of DT-treated mice (**Fig 3A**). Similarly, AP20187 treatment of MaFIA mice did not facilitate spread of VACV to the ovaries (**Fig 3B**). To directly examine the role of inflammatory macrophages in preventing the spread of VACV from the ear, we infected CCR2^-/-^ mice with VACV intradermally in the ear pinnae. In addition, we also infected mice lacking the chemokine receptor CX3CR1. A previous report has implicated CX3CR1^+^ DC in control of VACV following an intranasal infection [54]. However, neither CCR2^-/-^ nor CX3CR1^-/-^ mice demonstrated marked systemic spread of virus following dermal VACV infection (**Fig 3C**), indicating that, unlike in the intranasal infection model, locally recruited macrophage populations are unlikely to play a role in restricting VACV to the skin. We have previously described a requisite role for Ly6G^+^ cells in protection of the ear from tissue damage following VACV infection. To examine whether tissue protective Ly6G^+^ cells were required to prevent systemic dissemination of VACV we depleted these cells with anti-Ly6G antibody, which effectively depletes systemic and infiltrating Ly6G^+^ cells [32]. Similar to the MaFIA and LysMcre:iDTR mouse, both of which effectively deplete Ly6G^+^ cells at the site of VACV infection, we found that targeted depletion of Ly6G^+^ cells did not allow systemic spread of VACV (**Fig 3D**). Ly6G^+^ cells have been proposed to interact with CD8+ T cells at the site of infection to modulate local VACV replication in the ear [41]. Therefore we also examined the effect of depletion of Thy1^+^ T cells upon the spread of VACV from the ear. As with depletion of Ly6G^+^ cells, T cell depletion also failed to allow systemic spread of VACV (**Fig 3D**). Therefore, CLL treatment appears unique in its ability to facilitate systemic spread of VACV from a peripheral site of infection.

Macrophage populations have been implicated in the prevention of systemic disease and death following challenge with ECTV, the causative agent of mousepox [55]. In contrast to VACV, ECTV causes disseminated infection but is usually survived by C57BL/6 mice. After intradermal infection with ECTV, LysMcre:iDTR mice depleted with DT (**Fig 3E**) and MaFIA mice depleted with AP20187 (**Fig 3F**) both succumbed to lethal infection, similar to the phenotype we observed with CLL depletion [55]. However, CCR2^-/-^ mice were no more susceptible to ECTV mortality than wild-type mice (**Fig 3G**). Therefore, myeloid cell populations can be crucial for efficient immunity to poxvirus challenge, although the role that they play is likely different depending on the nature of the viral challenge, and potentially by the ability of a virus to replicate within myeloid cell populations.

### Lymph node macrophages do not restrict VACV spread

**Fig 4:**
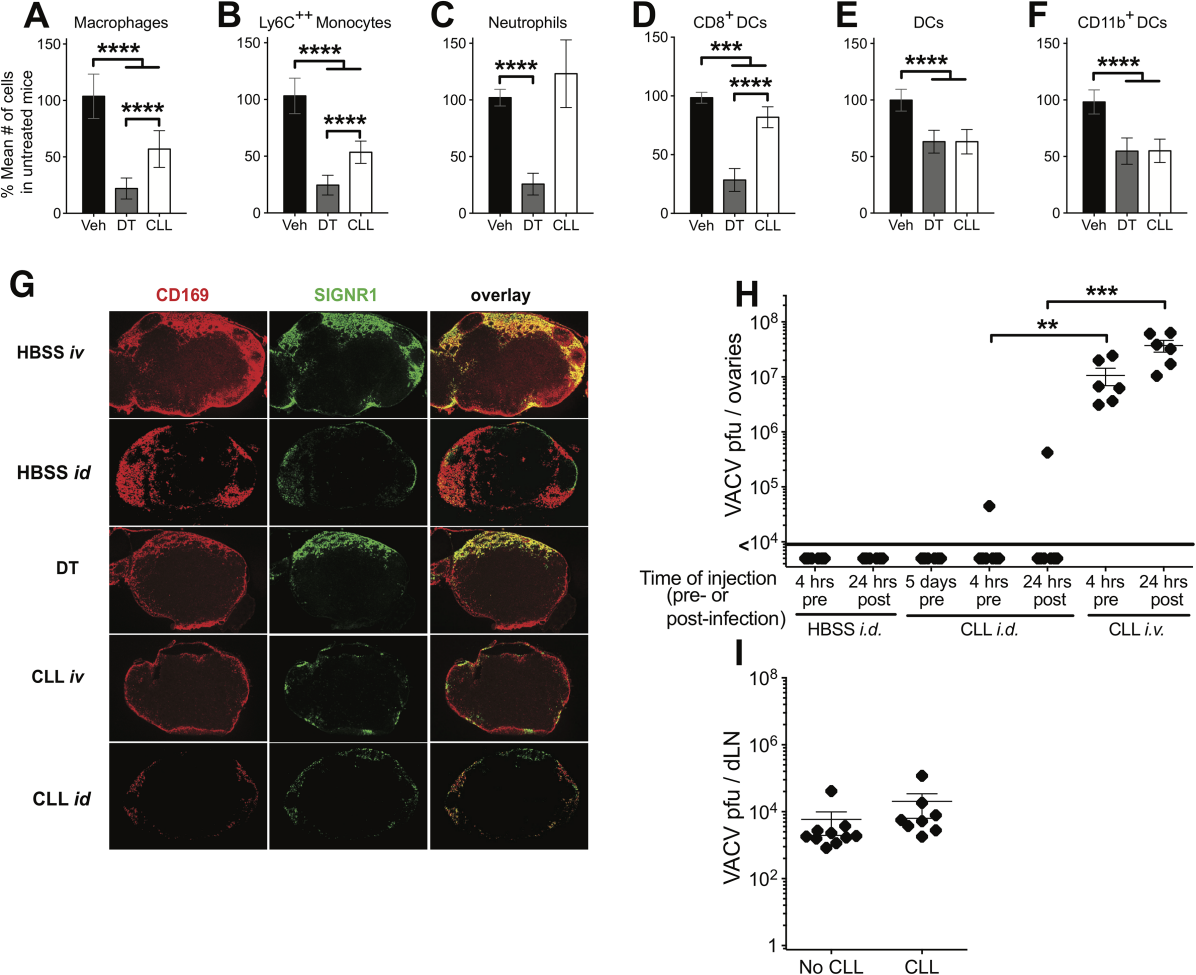
Draining lymph node macrophage populations do not prevent systemic spread of VACV. Naïve LysMcre:iDTR mice (n = 8–14 from >3 experiments) were given a single injection of CLL i.v., DT i.p., or vehicle i.v.. At 1 day post-depletion, cervical LN **(A-F)** were isolated and cells extracted. Flow cytometry was used to count the total number of (**A**) macrophage/monocytes (F4/80^+^ CD11c^-^ CD11b^+^ Ly6G^-^), (**B**) inflammatory monocytes (F4/80^-^ CD11c^-^ CD11b^+^ Ly6C^++^ Ly6G^-^), (**C**) neutrophils (F4/80^-^ CD11c^-^ CD11b^+^ Ly6C^+^ Ly6G^+^), (**D**) CD8^+^ DC (CD11c^+^ F4/80^-^ CD8^+^ CD11b^-^), (**E**) DC (CD11c^+^ F4/80^-^), and (**F**) CD11b^+^ DC (CD11c^+^ F4/80^-^ CD11b^+^ CD8^-^). (**A**-**F**) In order to compile data across many experiments data are expressed as % of the mean number of cells in untreated mice. Results include all data from a minimum of 3 independent experiments (n = 8–14). (**G**) Naïve LysMcre:iDTR mice (n = 3–4) were given a single injection of CLL i.v. or i.d., DT (100 ng/g) i.p., or vehicle i.v. or i.d.. At 1 day post-depletion, LN were isolated and flash-frozen in OCT compound. Then 10–12 micron sections were fixed using acetone and stained with antibodies to CD169 (red) and SIGNR1 (green). Results are representative of those from 3 independent experiments (n = 6). (**H**) C57BL/6 mice were infected intradermally with 10,000 pfu VACV in each ear pinna. Mice were given intradermal injections of CLL (25 μl/ear) or intravenous injections of CLL (250 μl/mouse) at the time shown pre- or post-infection. 5 days after infection, ovaries were harvested and used in a plaque assay for VACV titer. (**I**) Mice were given a single injection of CLL i.v. or vehicle and infected intradermally with 10,000 pfu VACV in each ear pinna. 5 days after infection, D-LN were harvested and used in a plaque assay for VACV titer. Results include all data from 3 independent experiments (n = 6–8).

Lymph node macrophages, in particular SCS macrophages, have a demonstrated role in restricting the spread of some viruses following peripheral infection [4–8]. Therefore, we examined depletion of myeloid cell populations in the D-LN following either a treatment that is permissive (systemic CLL) or non-permissive (DT treatment of LysMcre:iDTR) for VACV spread following intradermal infection, in an effort to find a uniquely depleted cell population to which we could attribute the function of restricting VACV spread. Naïve LysMcre:iDTR mice were given a single injection of CLL or DT, and myeloid cells in the D-LN were analyzed the following day (**Fig 4A–4F**). DT treatment reduced the populations of D-LN macrophages (**Fig 4A**), Ly6C^++^ monocytes (**Fig 4B**) neutrophils (**Fig 4C**), and CD8^+^ DC (**Fig 4D**) populations, as well as, to a lesser extent (<50%) bulk DC (**Fig 4E**) and CD11b^+^ DC populations (**Fig 4F**). Systemic CLL administration, which allows spread of VACV, depleted bulk DC (**Fig 4E**) and CD11b^+^ DC populations (**Fig 4F**) to a similar extent to the minor depletion with DT, which does not allow spread. However, although systemic CLL treatment did partially deplete D-LN macrophages (**Fig 4A**), Ly6C^++^ monocytes (**Fig 4B**), and CD8^+^ DC (**Fig 4D**) populations to a statistically significant degree, the depletion was never greater than 50%, i.e. DT was much more effective at depleting these populations than CLL. Therefore, there is no discernable population in the D-LN that is depleted more effectively by CLL, which allows disseminated infection, than by DT that does not allow a systemic infection to be established. These data indicate that it is unlikely that D-LN resident macrophages play an exclusive role in preventing dissemination of VACV following dermal infection.

Although systemic treatment with CLL failed to deplete myeloid cell populations in the D-LN, it has been published that local treatment with CLL can deplete SCS macrophages, thus allowing systemic dissemination of virus following peripheral vesicular stomatitis virus infection [4, 56]. Therefore, we examined the ability of systemic or local CLL administration, or DT treatment, to deplete CD169^+^ SCS macrophages or SIGN-R1^+^ medullary macrophages by microscopy. In vehicle-treated mice, CD169^+^ SCS macrophages form an intact barrier around the entire LN and SIGN-R1^+^ macrophages populate the medulla and are excluded from B and T cell areas (**Fig 4G**). DT treatment partially reduced CD169 staining, but systemic CLL did not deplete SCS macrophages to a discernable degree (**Fig 4G**). DT treatment also slightly reduced SIGN-R1 staining, and systemic CLL treatment increased depletion of medullary macrophages (**Fig 4G**). However, local administration of CLL greatly reduced staining of both CD169 and SIGN-R1, with the barrier around the LN clearly disrupted (**Fig 4G**). Therefore, we examined whether local CLL administration versus systemic CLL administration would allow dissemination of VACV to the ovaries 5 days post-infection. We injected CLL at the base of the ears on the day of infection or 1 day post-infection, timepoints at which an intravenous dose of CLL reliably permits VACV to spread. However, VACV was detected in the ovaries in only a small proportion of mice (2 of 14) in which CLL was delivered locally, compared to 100% of the mice given intravenous CLL in the same experiment (**Fig 4H**). Finally, we examined whether CLL treatment altered levels of VACV found in the D-LN at the peak of virus replication, on d5 post-infection. As previously published, levels of VACV in the D-LN were much lower than at the primary site of infection [27, 28], but were unaffected by systemic CLL treatment (**Fig 4I**). Taken together, these data support the conclusion that VACV drainage through the D-LN is unaffected by D-LN-resident myeloid cell populations, which are unlikely to play an exclusive role in preventing disseminated VACV infection.

### VACV infects splenic MZ and metallophilic MZ macrophages

**Fig 5:**
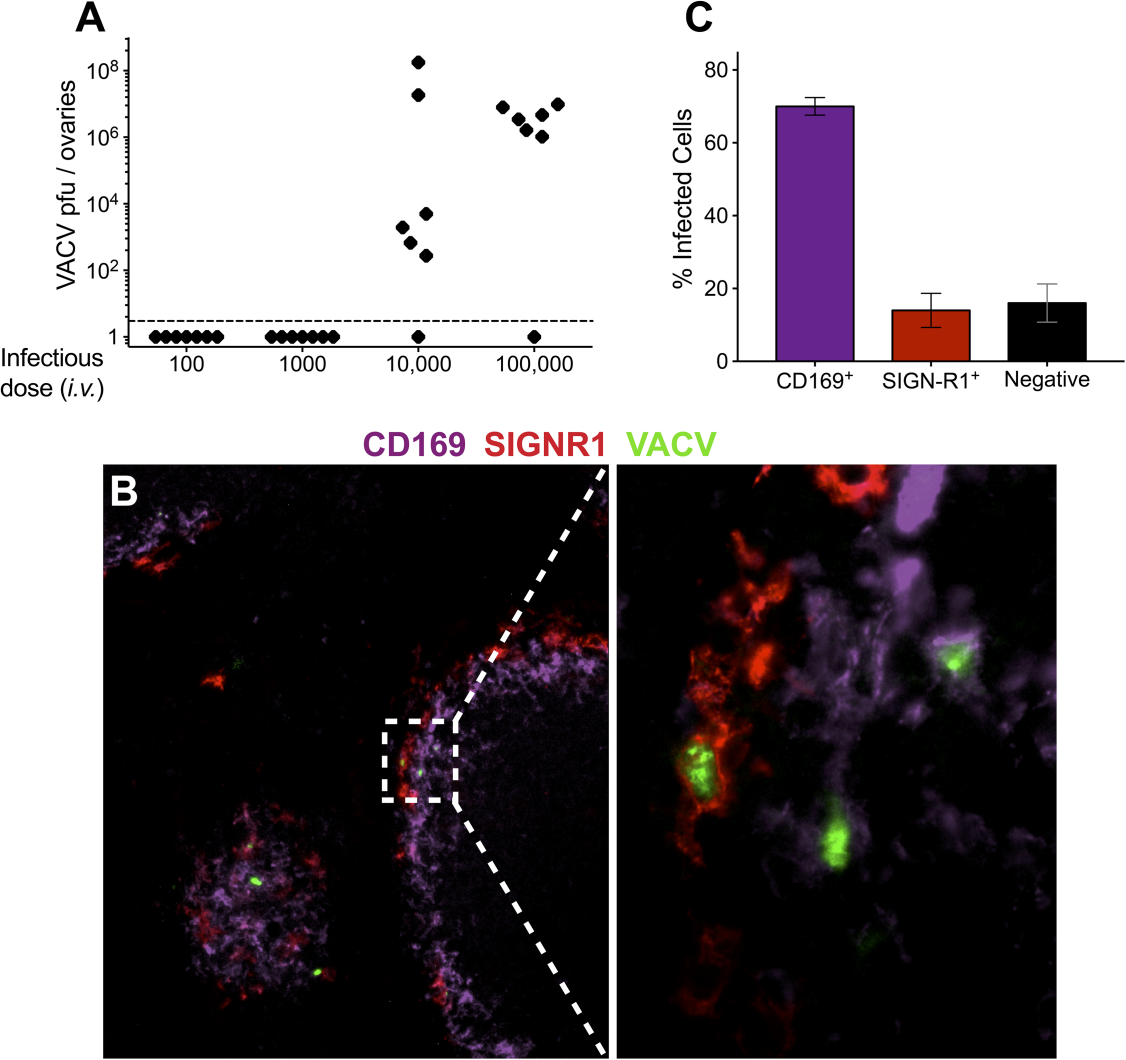
VACV infects splenic metallophilic MZ and MZ macrophages. **(A)** C57BL/6 mice were infected i.v. with 100–100,000 pfu VACV and ovaries were harvested and titered at 5 days post-infection. **(B)** C57BL/6 mice were infected i.v. with 10,000 pfu VACV-GFP. Spleens were harvested at 6 hours post infection and flash-frozen in OCT compound. Then 10–12μm sections were fixed and stained with antibodies to CD169 (purple) and SIGNR1 (red). **(C)** Quantification of fluorescent microscopy of infected cell populations in the spleen at 6hr post infection with VACV-GFP. Results are representative of those from 3 independent experiments (n = 6).

Our data support the model that VACV moves through the lymph node and enters the blood stream, so we sought to mimic these conditions and examine the role of systemic macrophage populations in control of VACV dissemination. Intradermal infection inoculates 2x10^4^ pfu per mouse, not all of which stays in the skin. In previous studies, we found that less than half of the initial inoculum is recovered from the infected ear at 1 day post-infection [57], suggesting that a considerable dose of VACV reaches the lymphatics and circulation. This could explain the occasional establishment of disseminated infection even in mice with intact phagocytes. When 10^5^ pfu VACV was injected *i*.*v*., ~ 85% of mice experienced uncontrolled viral replication in the ovaries 5 days later (**Fig 5A**). However, when the inoculum was reduced to 1000 pfu VACV in the same injection volume, the virus never established infection in the ovaries in multiple experiments (**Fig 5A**). Infection with 1000 pfu *i*.*v*. may lead to fewer viral particles reaching the circulation than *i*.*d*. infection with 2x10^4^ pfu, so this finding demonstrates that dissemination of low levels of VACV in the blood, similar to those moving from a site of dermal infection, was blocked.

To ascertain whether systemic macrophages become infected with VACV-GFP in order to block dissemination, we inoculated mice with 10^5^ pfu VACV *i*.*v*. and examined which cells in the spleen were infected 24 hours post-infection. We only observed infected GFP^+^ cells in the MZ of the spleen, the area known to filter the blood (**Fig 5B**). Of the infected cells, the majority (~70%) stained for CD169, denoting the metallophilic MZ macrophage population (**Fig 5B and 5C**) [58]. A smaller population (~15%) of SIGN-R1^+^ MZ macrophages was also infected. Therefore, it is possible that these MZ macrophage populations can “soak up” VACV in the circulation to prevent subsequent spread.

### Systemic “Filtering” macrophages are preferentially ablated by CLL treatment

**Fig 6:**
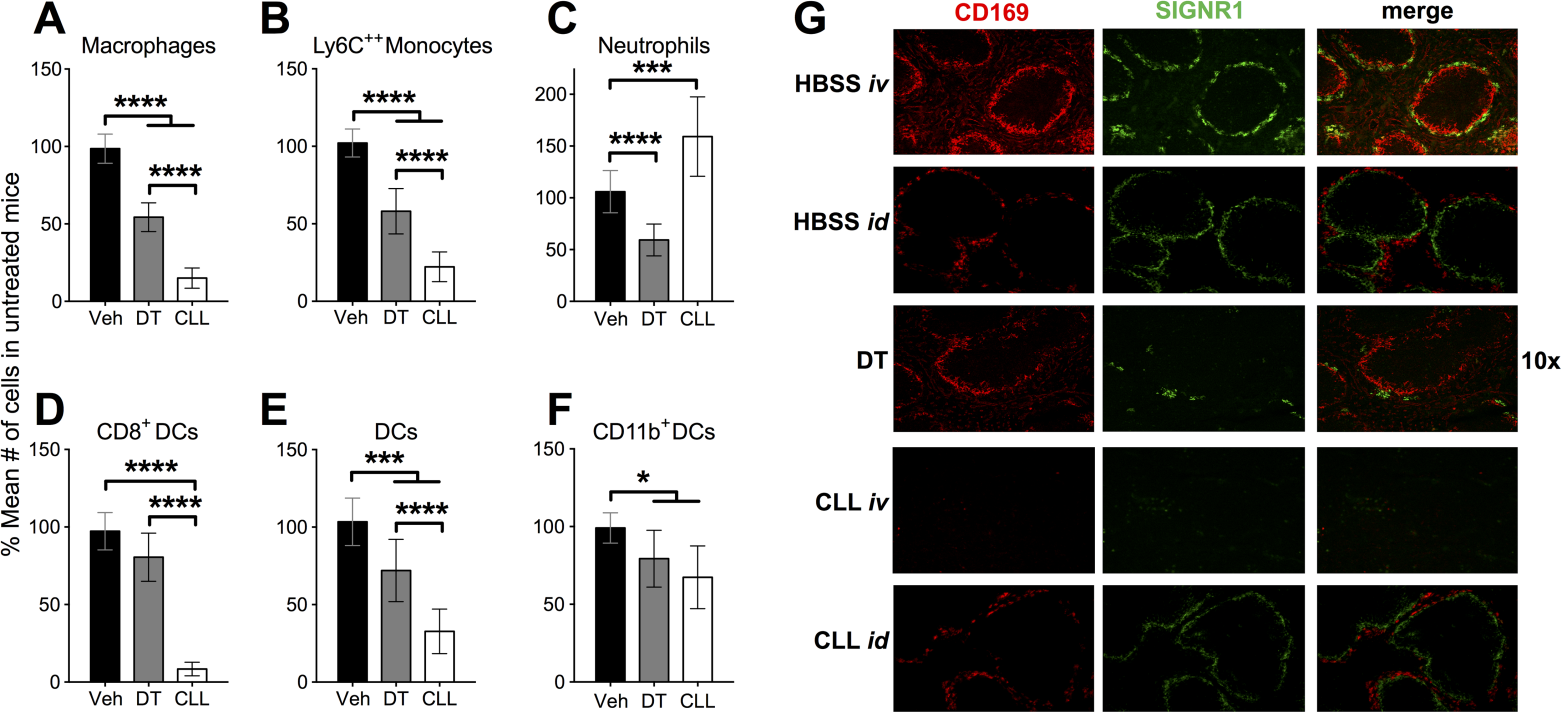
Splenic MZ macrophages are only depleted by conditions that allow systemic spread of VACV. Naïve LysMcre:iDTR mice (n = 8–12 from >3 experiments) were given a single injection of CLL i.v., DT i.p., or vehicle i.v.. At 1 day post-depletion, spleens **(A-F)** were isolated and cells extracted. Flow cytometry was used to count the total number of (**A**) macrophage/monocytes (F4/80^+^ CD11c^-^ CD11b^+^ Ly6G^-^), (**B**) inflammatory monocytes (F4/80^-^ CD11c^-^ CD11b^+^ Ly6C^++^ Ly6G^-^), (**C**) neutrophils (F4/80^-^ CD11c^-^ CD11b^+^ Ly6C^+^ Ly6G^+^), (**D**) CD8^+^ DC (CD11c^+^ F4/80^-^ CD8^+^ CD11b^-^), (**E**) DC (CD11c^+^ F4/80^-^), and (**F**) CD11b^+^ DC (CD11c^+^ F4/80^-^ CD11b^+^ CD8^-^). (**A**-**F**) In order to compile data across many experiments data are expressed as % of the mean number of cells in untreated mice. Results include all data from a minimum of 3 independent experiments (n = 8–14). (**G**) Naïve LysMcre:iDTR mice (n = 3–4) were given a single injection of CLL i.v. or i.d., DT i.p., or vehicle i.v. or i.d.. At 1 day post-depletion, LN were isolated and flash-frozen in OCT compound. Then 10–12 micron sections were fixed using acetone and stained with antibodies to CD169 (red) and SIGNR1 (green). Results are representative of those from 3 independent experiments (n = 8).

Many resident tissue macrophage populations can “filter” the blood of particles, and splenic MZ macrophages have a demonstrated role in restricting the spread of some viruses following systemic infection [11–18]. Therefore, as a model for the role of systemic macrophages that filter the blood, we examined depletion of splenic myeloid cell populations in order to find a splenocyte population depleted by CLL, but not by DT treatment of LysMcre:iDTR mice, to which we could attribute the function of restricting VACV spread. When spleens of LysMcre:iDTR mice were analyzed following CLL or DT treatment, we found that systemic CLL treatment was markedly better at ablating splenic myeloid cell populations than DT (**Fig 6A–6F**). DT treatment reduced the numbers of macrophages (**Fig 6A**), monocytes (**Fig 6B**), bulk DC (**Fig 6E**) and CD11b^+^ DC (**Fig 6F**) in the spleen significantly vs. controls, but depletion was never greater than 50%. In contrast, CLL depleted populations of macrophages (**Fig 6A**), monocytes (**Fig 6B**) and CD8^+^ DC (**Fig 6D**) by ~85%, and populations of bulk DC by ~70% of control levels (**Fig 6E**). Depletion of CD11b^+^ DC was similar with DT or CLL treatment (**Fig 6F**) and, although DT treatment reduced the number of neutrophils, the permissive CLL treatment actually increased numbers of these cells (**Fig 6C**), which supported our observation that systemic depletion of these cells with anti-Ly6G antibody did not allow systemic spread of VACV (**Fig 3D**). From these data, it is clear that DT treatment does not deplete the majority of a number of myeloid cell populations in the spleen, several of which are almost ablated by CLL treatment.

We sought to confirm that the populations of MZ macrophages that become infected with VACV are depleted by permissive systemic CLL treatment versus the non-permissive treatments, namely dermal CLL treatment and DT treatment of LysMcre:iDTR mice. We isolated spleens from treated or control mice 5 days post-depletion with DT or CLL, and stained cryosections with antibodies to CD169 and SIGNR1. Treatment with DT reduced staining with anti-SIGN-R1, indicating depletion of MZ macrophages. However, staining with anti-CD169 was only marginally reduced, indicating that the metallophilic MZ macrophages, which are the primary target of VACV in the spleen, remain in DT treated mice (**Fig 6G**). These results are consistent with the flow cytometry data, which show a ~45% reduction in the number of macrophages in the spleen of DT treated mice. In contrast, systemic, but not local, treatment with CLL completely ablated staining with both antibodies (**Fig 6G**), indicating ablation of both populations of MZ macrophages. This is consistent with the ~90% reduction in the number of macrophages in the spleen of CLL treated mice. Together with the flow cytometry analysis, these data indicated a markedly enhanced ability of the permissive systemic CLL treatment to deplete splenic macrophages, particularly metallophilic MZ macrophages, when compared to the non-permissive DT and local CLL treatments.

### Systemic macrophages, but not DC, are crucial to block VACV dissemination that bypasses the D-LN

**Fig 7:**
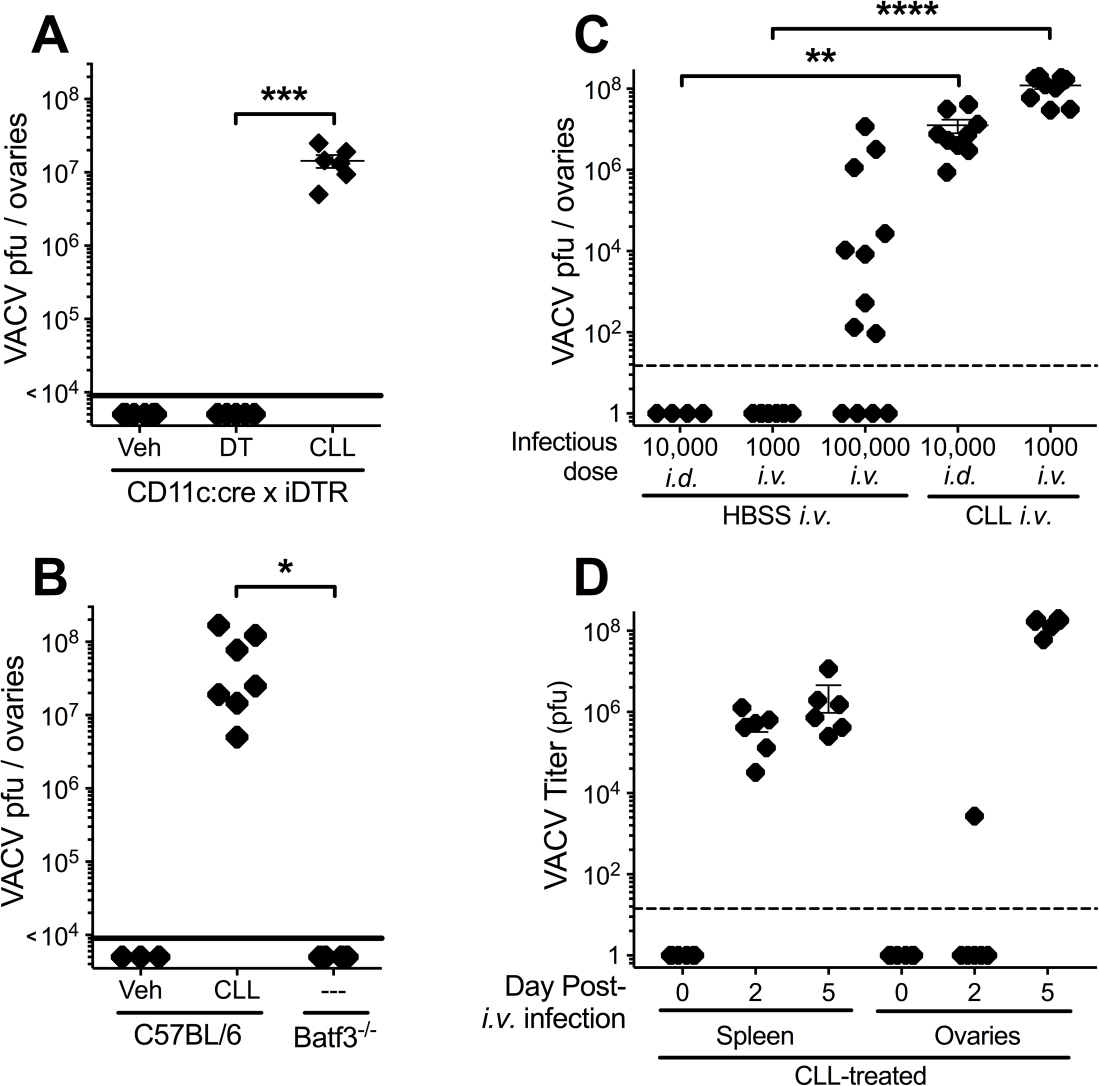
Systemic macrophages, but not DC, are crucial for blocking VACV dissemination during infection that bypasses the D-LN. (**A–C**) Mice were treated as follows and at 5 days post-infection, ovaries were harvested and used in a plaque assay for VACV titer. (**A**) Naïve CD11ccre:iDTR mice (n = 8–10) were given a single injection of CLL i.v., DT (20 ng/g) i.p., or vehicle i.v. 24 hours pre-infection. **(B)** C57BL/6 or Batf3^-/-^ mice were infected i.v. with 1000 pfu VACV. As a positive control, half the C57BL/6 mice were pre-depleted with CLL i.v. (n = 6–10) **(C)** C57BL/6 mice were infected with 1000, 10,000 or 100,000 pfu VACV i.v. or i.d.. 24 hours pre-infection, mice were injected with 250 μl CLL or HBSS i.v.. (**D**) Mice were pre-depleted with CLL i.v. and infected i.v. with 1000 pfu VACV, then spleen or ovaries were harvested at 0, 2 or 5 days post infection and a plaque assay used to determine VACV titer.

We have previously demonstrated that DC populations, primarily plasmacytoid DC and CD8^+^ DC, are essential for mice to survive ECTV infection [50]. Others have also shown that recognition of ECTV infection by DC is essential [59]. DC were sensitive to CLL depletion and, to a much lesser extent, to DT depletion (**Fig 6E**). Therefore, we examined the spread of VACV to the ovaries after a dermal infection of mice in which DC were depleted. We treated CD11ccre:iDTR mice with DT using a regimen we have previously characterized during ECTV infection, and which confers lethal susceptibility to ECTV infection [50]. DT treatment of CD11ccre:iDTR mice failed to allow spread of VACV following dermal infection (**Fig 7A**). The CD11b^+^ DC subpopulation was depleted by DT and CLL to a similar extent (**Fig 6F**), so it is unlikely that this cell population is required to prevent the spread of VACV. However, CD8^+^ DC were the major DC population depleted by CLL treatment, but not signficiantly by DT treatment (**Fig 6D**), so we used VACV to intradermally infect Batf3^-/-^ mice, which lack CD8^+^ DC [60]. However, as seen in **Fig 7B**, VACV infection did not disseminate in Batf3^-/-^ mice. This indicates that it is not DC, nor the specialized splenic CD8^+^ DC population that restricts dissemination of VACV.

Next, we examined whether the monocyte/macrophage populations that are preferentially depleted by CLL treatment prevented the ability of small quantities of VACV in the bloodstream to spread systemically. To ensure that we were examining the clearance of bloodborne virions by systemic macrophage populations, we inoculated mice with either high (10^5^ pfu) or low (10^3^) dose VACV intravenously, or with 10^4^ pfu *i*.*d*., as in our previous experiments. The high dose VACV *i*.*v*. overcame the immune system and spread to the ovaries in the majority of mice, but both low dose *i*.*v*. and *i*.*d*. VACV never reached the ovaries (**Fig 7C**). However, when mice were pre-depleted with CLL, infection with 1000 pfu *i*.*v*. or 10^4^ pfu *i*.*d*. resulted in the spread of equally high levels of VACV to the ovaries 5 days post-infection (**Fig 7C**). Therefore, CLL-depleted cells, likely systemic macrophages that filter blood, are essential to prevent disseminated VACV infection.

Finally, we examined whether systemic macrophages can impair the dissemination of VACV prior to the virus reaching the ovaries. To do so, we measured virus titers in a “filtering organ”, the spleen, versus the target organ (the ovaries) at various times after infection of CLL-depleted mice with 1000pfu VACV *i*.*v*.. On the day of infection titers from each organ were undetectable, but by 2 days post-infection titers in the spleens of all mice approached 10^6^ pfu (**Fig 7D**). In contrast, only one of eight VACV-infected mice showed detectable titers in the ovaries 2 days after infection (**Fig 7D**). By 5 days post infection, however, all the CLL-depleted mice showed levels of VACV in the ovaries that surpassed those in the spleen, where titers appear to have reached a plateau soon after 2 days post-infection (**Fig 7D**). These results are consistent with VACV in the bloodstream being filtered by systemic macrophages prior to spread of VACV to the target organ, the ovaries.

## Discussion

Many viruses of importance to human health such as variola (smallpox), monkeypox, polio, coxsackie, rubella, yellow fever, dengue, West Nile, mumps, measles, varicella (chickenpox), lymphocytic choriomeningitis virus, vesicular stomatitis virus, herpes simplex 1, and many more, penetrate their hosts through disruptions of epithelial surfaces and disseminate stepwise to distant organs following a lympho-hematogenous route [1, 2, 61]. However, the majority of studies examining the immune response to viruses that infect in this manner focus on immunity following *i*.*v*. or *i*.*p*. infection. Following these routes of infection, the virus has access to populations of cells that it may not normally encounter upon infection via a natural route. The geographical restriction of infection to a single site is an important factor in shaping the nature of the subsequent response. In this study, we examined the role of myeloid cells at three distinct spatial checkpoints, namely: 1) the site of infection, 2) the draining lymph node, and 3) systemically by organs that filter the blood, in control of VACV systemic dissemination. We find that local myeloid cell populations do not play an exclusive role in preventing disseminated VACV infection and, surprisingly, that D-LN resident macrophage populations are not the only point of control in restricting the spread of VACV. Rather, our data supports a model in which VACV spreads from the site continually for 4 days post-infection, and virus can move past the D-LN, into the bloodstream where further spread is prevented by systemic macrophage populations with access to the bloodstream.

The prevailing theory at present is that the major spatial checkpoint in preventing the spread of virus after a peripheral infection resides with the SCS macrophages in the D-LN [4–7] or, in the absence of SCS macrophages, the medullary macrophages of the D-LN [8]. We have previously demonstrated that SCS macrophages and other myeloid cell populations in the D-LN are infected with VACV within hours following dermal infection [34]. Thus, VACV spreads via the lymphohematogenous route (via the lymph node into the blood). However, here our data clearly show D-LN macrophage populations were not necessary to prevent systemic dissemination of VACV. The most compelling evidence that D-LN macrophages have a vital role in preventing spread of peripheral virus infections comes from administration of CLL locally following infection with VSV [4], MVA [62], a Rabies virus-based vector [63] or MCMV [7], implicating macrophage populations in the D-LN as requisite to prevent virus spread or initiate an adaptive immune response. However, local administration of CLL failed to confer the ability of VACV to spread systemically to the ovaries, indicating that the D-LN is not a vital checkpoint in the prevention of VACV spread. The fundamental difference between our observation and those with other viruses could be explained by the use of replication deficient viruses in a number of the previous publications [4, 62, 63]. Under conditions where the immune system only has to control spread of input loads of virus, the D-LN macrophage populations are likely to be able to internalize sufficient virus to prevent spread. In contrast, our full replicative virulent Western Reserve VACV can produce up to 10^9^ pfu (from 10^4^ pfu input) in the ear at the peak of replication [57] and the D-LN macrophages are likely consistently saturated with high concentrations of virions. Alternatively, the proposal from Moseman *et al* that the role of CLL-depleted SCS macrophages is to become infected and produce Type-I IFN [56] may indicate why VACV spread is not prevented by D-LN macrophages. All of the viruses for which D-LN macrophages have been described to prevent systemic spread are very sensitive to Type-I IFNs. However, VACV encodes a significant number of immunomodulatory molecules that block Type I IFN induction, action or signaling [64], and we have previously found that dermal VACV infection poorly induces Type I IFN production [57]. Indeed, VACV infection can restore the ability of VSV to replicate in the presence of IFN [65]. In contrast, MVA, a non-replicating attenuated vector derived from VACV, lacks many of the genes that modulate Type-I IFN [66]; this increased IFN-sensitivity, combined with reduced replicative ability, may account for an enhanced role for D-LN macrophages in MVA infection. Highly virulent viruses that express numerous mechanisms of evading the Type-I IFN response, such as VACV or ECTV, may bypass D-LN macrophages on their way to establishing a systemic infection.

It is often thought that one of the major roles of the innate immune response is to restrict replication of a pathogen at the original site of infection prior to the recruitment of adaptive immune cells that can then eliminate the infection. After dermal VACV infection, replicating virus is constrained at the site of infection, most often the ear pinnae [28]. This route mimics the natural route of infection with the majority of poxviruses, and also the major route of immunization used during smallpox vaccination or with many VACV-based vectors. Virus enters the dermis, stimulating resident somatic and immune cell populations to recruit neutrophils and monocytes/macrophages that can attack the pathogen [31, 32], and also triggering DC migration away from the area of infection to present antigen to naïve T cells [38–40]. Multiphoton microscopy studies have revealed that a “granuloma-like” structure forms following dermal VACV infection, with a virus infected core containing monocytes and other myeloid cell populations surrounded by a layer of adoptively-transferred CD8^+^ T cells that kill infected cells leaving the core [41]. However, we have shown that CD8^+^ T cells do not begin to accumulate at the site of infection prior to day 5 post-infection [32]. Here we show that the initial VACV inoculum and virus produced prior to day 4 post-infection spreads systemically when mice are treated with CLL, but after that time point the virus remains restricted to the skin. Therefore, it appears that the anatomy of the innate and adaptive response prevents VACV spread from the initial site of infection only at 4 or more days post-infection.

The identity of the cell population(s) that are required to prevent VACV dissemination is unknown. Hickman *et al* postulated that CD8^+^ T cell killing of infected monocytes leaving the viral lesion in the skin prevents virus dissemination [41]. However, replication of VACV in monocytes was extremely low (<2 pfu/cell) [41], and continual depletion of T cells did not allow spread of virus to the ovaries (**Fig 3D**). Infiltrating Ly6G^+^ myeloid cells are also not required to prevent VACV dissemination ([32] and **Fig 3D**), although depletion of both CD8^+^ T cells and Ly6G^+^ myeloid cells did increase local replication dramatically [41]. Indeed, the only treatment described to allow VACV dissemination following dermal challenge with the low doses of VACV we use here is CLL administration [32]. Therefore, before we began this study we presumed that CLL-depleted monocyte/macrophages at the site of infection acted locally to prevent VACV dissemination. However, macrophages at the site of infection do not exclusively restrict VACV spread as neither blockade of the recruitment of monocyte/macrophages (in CCR2-deficient mice), nor depletion of bulk local myeloid cells (via in LysMcre:iDTR or MAFIA mice), allowed VACV spread following dermal infection.

Although monocyte/macrophages at the site of infection do not appear to restrict VACV spread, they do appear to have a dramatic impact upon local pathogenesis and tissue damage. Despite only a minor impact on local VACV replication (1.5–3 fold [32]), we have shown that local macrophages do control the lesion size at the site of infection. This is likely a role of recruited monocyte/macrophage populations, as the phenotype of CLL-treated mice is reproduced in CCR2^-/-^ mice that lack recruitment of inflammatory monocytes. Besides a role for macrophages following VACV infection, the simultaneous depletion of Ly6G^+^ cells and CD8^+^ T cells demonstrated that local VACV replication in the ear is not self-limiting, but is controlled by innate and adaptive effector cells acting in concert [41]. Dual depletion strategies examining depletion of macrophages along with depletion of other cell types, such as Ly6G^+^ cells or CD8^+^ T cells, have not been explored, but may reveal a cooperative effect on the control of local VACV replication. The mechanisms used by recruited monocyte populations to control the lesion size are also unknown, but are displayed at 7–8 days post-infection, a time point significantly after monocyte infiltration has peaked [32]. Ly6G^+^ myeloid cells, acting through a reactive oxygen species (ROS)-dependent mechanism, limit the extent of tissue damage, offering the possibility that Ly6G^+^ myeloid cells and recruited monocytes act in concert to accomplish this task. Whether this is via enhanced ROS production, or production of skin specific wound healing factors such as IL-22, remains a focus of ongoing investigation.

Our data clearly demonstrate that systemic CLL-depleted populations are required to prevent further VACV dissemination once the virus has entered the bloodstream. It is currently not technically feasible to deplete individual macrophage populations in particular organs. Multiple populations in secondary lymphoid organs or non-lymphoid organs may be required to prevent VACV dissemination. Indeed, it is possible that myeloid cells at the site of infection, in the D-LN, and systemically, all act together to prevent fulminant infection. If we administer small doses of VACV to bypass the role of the D-LN, metallophilic MZ and MZ macrophages in the spleen quickly become VACV-infected, as they do with numerous other bloodborne viruses [11–18]. CLL-depleted macrophages, such as metallophilic MZ macrophages in the spleen (which are not depleted by DT), Kupffer cells in the liver or juxtaglomerular macrophages of the kidney, may play a role in both preventing VACV spread to the ovaries and protection of the spleen from ongoing VACV replication. Non-productive infection of “suicide macrophages” that filter either lymph or the blood has been proposed as a means to reduce virus spread [4, 15], and may be the mechanism that allows protection of the ovaries. VACV replicates poorly, if at all, in monocyte/macrophages, in contrast to ECTV which replicates rapidly and effectively in macrophages and DC. The different capabilities of these related poxviruses to replicate within macrophage populations may explain why ECTV can spread rapidly and cause death in susceptible mice. Indeed, following ECTV infection it is the DC populations, rather than macrophages, that are required for survival [50, 59] and this may reflect an ability to replicate within macrophages. Therefore, our data supports VACV infection of “suicide macrophages” in organs that filter the blood, thus removing VACV virions from the circulation and reducing dissemination. If this is the case, the applicability of these findings may be limited to viruses that do not replicate effectively in macrophage populations. Alternatively, infection of populations of systemic macrophages may be important for production of Type-I interferon [18, 67], IL-1 [14] or induction of T cell [16] or antibody [5] responses. Under these circumstances, the applicability of the data shown here may be much broader. However, it is not technically possible to deplete these subsets specifically in a single organ at this time so a full description of the role of the required CLL-depleted cell type is not possible to delineate.

It has recently become clear that the myeloid cell compartment is exemplified by its plasticity, and that the local environment dramatically alters the phenotype and function of cells [68, 69]. In the context of our own studies, it is clear that macrophages present at the infection site, in the D-LN or at distal sites, have dramatically different roles during VACV infection. Therefore, it is no longer appropriate to just assign a role to “macrophages”, and we await the technology to study the role of individual phenotypic and spatially situated populations. Our studies also demonstrate that even injection of relatively low levels of virus that appear to replicate only locally can lead to systemic distribution of virions prior to initiation of an adaptive immune response. Normally these systemically distributed virions are contained by the innate immune response, in this case, by macrophage populations. However, if large doses of virus are inoculated systemically, the role of the innate response is not revealed. Therefore, it is essential to infect via natural routes with relevant doses of virus in order to fully elucidate the breadth of any immune response during virus infection and thereby gain insight into potential checkpoints that may be manipulated clinically.

## Materials and methods

### Mice

C57BL/6 mice were purchased from Charles River Laboratories. CCR2^−/−^ (catalog no. 004999), MaFIA (catalog no. 005070), Batf3^-/-^ (catalog no. 013755), CX3CR1^gfp/gfp^ (catalog no. 005582), LysM-cre (catalog no. 004781), CD11c-cre (catalog no. 008068) and iDTR (catalog no. 007900) mice were purchased from Jackson Laboratory and subsequently bred at the Hershey Medical Center. LysMcre:iDTR and CD11ccre:iDTR mice were heterozygotes derived from crossing purebred LysM-cre or heterozygous CD11ccre with purebred iDTR mice. All knockout mouse strains were on the C57BL/6 background after a minimum of 12 backcrosses.

### Ethics statement

All animals were maintained in the specific-pathogen-free facility of the Hershey Medical Center and treated in accordance with the National Institutes of Health and AAALAC International regulations. All animal experiments and procedures were approved by the Penn State Hershey IACUC (Animal Welfare Assurance # A3045-01) that follows the Office of Laboratory Animal Welfare PHS Policy on Humane Care and Use of Laboratory Animals, 2015.

### Murine viral challenge

VACV (strain Western Reserve) stocks were produced in 143B TK^−^ cell (American Type Culture Collection, ATCC) monolayers and ECTV (strain Moscow) stocks were produced in L929 cell (ATCC) monolayers [70]. VACV was further purified by ultracentrifugation through a 45% sucrose cushion. For intradermal infection with VACV, mice were anesthetized using ketamine-xylazine and injected in each ear pinna with 10^4^ pfu in a volume of 10 μl [28]. For intravenous (*i*.*v*.) infection with VACV, mice were injected in the tail vein with the dose shown in a volume of 400 μl. For intradermal infection with ECTV, mice were injected in the right rear footpad with 3,000pfu. For infection via scarification, a droplet of 10^6^ pfu was placed on the ear and the ear scratched 20x through the droplet of virus with a 27 gauge needle. The droplet of virus was then removed.

To assess dermal pathogenesis, ear thickness was measured using a 0.0001-m. dial micrometer (Mitutoyo), and lesion progression was measured using a ruler [32]. To analyze the presence of replicating virus, organs were harvested, subjected to three freeze-thaw cycles in HBSS, ground in a Dounce homogenizer, and sonicated prior to a conventional plaque assay [32].

### Cell depletion

To deplete phagocytes, mice were injected *i*.*v*. with doses of 200–250 μl clodronate-loaded liposomes (CLL) in PBS, or 25 μl *i*.*d* in the center of the ear pinnae. Cl_2_MDP (or clodronate) was from Roche Diagnostics GmbH, Mannheim, Germany. Liposomes were prepared using Phosphatidylcholine (LIPOID E PC, Lipoid GmbH) and cholesterol (Sigma) [71]. For depletion of CD115-expressing cells, MaFIA mice were injected intraperitoneally (*i*.*p*.) with AP20187 (kind gift of Ariad Pharmaceuticals), diluted in sterile water containing 4% ethanol, 10% PEG-400, and 1.7% Tween immediately before injection. We followed the prescribed MaFIA injection regimen of 10 μg/g AP20187 on days -4, -3, -2, and -1 pre-infection, and 1 μg/g AP20187 on the day of infection and every third day thereafter [32]. For depletion of tissue-resident CD115-expressing cells, mice were injected *i*.*p*. with 50 μg/g AFS98 in HBSS/0.1%BSA on days -4, -2, and 0 pre-infection. For depletion of LysM-expressing cells, LysMcre:iDTR mice were injected *i*.*p*. with diphtheria toxin (DT) (Sigma D0564), diluted in PBS. For a single depletion, mice were injected with 100 ng/g DT. Sustained depletion involved multiple injections of 40 ng/g DT.

### Flow cytometry

VACV-infected ear pinna were cut into strips and digested in 1 mg/ml collagenase XI (Sigma C7657) for 60 min at 37°C. Spleens and LN were minced and digested in 1 mg/ml collagenase D (Roche) for 40 min at 37°C. Live cells were blocked and stained on ice in 2.4G2 cell supernatant containing 10% normal mouse serum (Gemini Bio-Products 100–113). To stain cells for flow cytometry we used antibodies to F4/80 (clone BM8) and B220 (RA3-6B2) from eBioscience, Ly6G (clone 1A8) and CD45 (30-F11) from BioLegend, and Ly6C (clone AL-21), CD45.2 (104), CD11c (HL3), CD8α (53–6.7), CD11b (M1/70), CD19 (1D3), CD90.2 (53–2.1), and NK1.1 (PK136) from BD. CD19, CD90.2 and NK1.1 antibodies were all labeled with biotin to aid in gating out lymphocytes. Phycoerythrin (PE)-Cy7-streptavidin (BD) was used to label biotin-conjugated antibodies. Sample acquisition was performed with an LSRII or Fortessa flow cytometer (BD), and data were analyzed with FlowJo software (TreeStar). In order to compile data across many experiments, data are expressed as % of the mean number of cells in untreated mice.

### Immunofluorescence microscopy

Spleens and LN from naïve mice were harvested and embedded in Tissue-Tek OCT (Sakura Finetek), then rapidly frozen by immersion in liquid nitrogen-cooled 2-methyl butane, and kept at -80°C overnight. Cryostat sections (10–12 μm) were cut at -20°C, mounted on glass slides, air-dried for 2–3 hours, fixed for 10–15 minutes in cold acetone, air-dried again for 30 minutes, and stored at -80°C. Slides were warmed to room temperature and stained in 1x TBS with 5% BSA and 0.1% Tween 20. Sections were stained with antibodies to SIGNR1 (22D1), secondary labeled with mouse anti-hamster Alexa Fluor 488 (1:100 dilution). CD169 was visualized with the clone MOMA-1 (Abcam), biotinylated and secondary labeled with streptavidin-PE (1:200 dilution). Microscope and software used to analyze microscopy were from Leica Microsystems (Buffalo Grove, IL).

To visualize infection of systemic macrophages with VACV we infected mice with 10,000 pfu VACV-GFP (strain Western Reserve, with no gene deletions). Six hours later spleens were harvested, flash frozen, sectioned (10 μm) and fixed in 2% paraformaldehyde in PBS (pH 7.4). Sections were then stained with antibodies to CD169 and SIGN-R1 as above.

### Statistics

Data were graphed and analyzed using Prism software (Graphpad). All data are expressed as mean ± standard error of mean. Means were compared using either an unpaired students t-test or two-way ANOVA as applicable. Survival curves were analyzed using a Log-rank test. Significance between groups was determined by p-value below 0.05, and is displayed as; * = p<0.05; ** = p<0.01; *** = p<0.001.
